# Relative age and ADHD symptoms, diagnosis and medication: a systematic review

**DOI:** 10.1007/s00787-018-1229-6

**Published:** 2018-10-06

**Authors:** Josephine Holland, Kapil Sayal

**Affiliations:** Division of Psychiatry and Applied Psychology, School of Medicine, CANDAL (Centre for ADHD and Neurodevelopmental Disorders across the Lifespan), Institute of Mental Health, University of Nottingham, Nottingham, UK

**Keywords:** ADHD, Relative age

## Abstract

There is a growing international literature investigating the relationship between attention-deficit/hyperactivity disorder (ADHD) and younger relative age within the school year, but results have been mixed. There are no published systematic reviews on this topic. This study aimed to systematically review the published studies on the relative age effect in ADHD. Systematic database searches of: Medline, Embase, PsycINFO, Web of Science, ERIC, Psychology and Behavioral Sciences Collection and The Cochrane Library were conducted. Studies were selected which investigated the relative age effect in ADHD in children and adolescents. Twenty papers were included in the review. Sixteen (of 20) papers reported a significantly higher proportion of relatively younger children being diagnosed with ADHD and/or receiving medication for this. Meta-analyses involving 17 of these 20 papers revealed a modest relative age effect in countries with higher prescribing rates, risk ratio = 1.27 (95% CI 1.19–1.35) for receipt of medication. The relative age effect is well demonstrated in countries with known higher prescribing rates. Amongst other countries, there is also increasing evidence for the relative age effect, however, there is high heterogeneity amongst studies. Further research is needed to understand the possible reasons under-pinning the relative age effect and to inform attempts to reduce it.

## Introduction

Attention-deficit/hyperactivity disorder (ADHD) is a common childhood neuro-developmental disorder, characterised by three core symptoms: inattention, hyperactivity and impulsiveness causing an impairment in functioning [1]. Although epidemiological studies suggest that globally ADHD affects around 5% of school-age children, diagnosis and prescription rates are heterogeneous between countries [2–7] and estimated prescribing rates vary, for example, from 0.9% in Denmark to 4.6–4.7% in Canada and Iceland [8].

The receipt of a clinical diagnosis of ADHD depends on evidence of symptoms affecting functioning in more than one setting, for example, both at school and at home. The diagnostic process, therefore, usually involves the collection of information from those who encounter the child in different contexts, for example, the child’s parents and school teachers, as well as the observations and interpretation of the health care professionals conducting the assessment [1].

There is an overwhelming literature documenting neurobiological, clinical and pharmacological evidence for the validity of ADHD as a diagnosis [9]. Despite the operationalization of the diagnostic process, since diagnosis involves clinical judgment, without an objective test, there remain a number of areas of debate within the literature [10, 11]. This systematic review focuses on one debate, whether relative age within the academic year affects the likelihood of a child being diagnosed with and/or receiving medication for ADHD. In many countries, there is a set age at which a child starts their first year of school, with a chronological date cut-off, e.g. 1st of September. This means that one child, born early in September may be 5 years old when the academic year starts, however, a child born at the end of August will only recently have turned 4 years of age. It would be expected that the older child will be more developmentally mature than the younger child, however, the academic and developmental expectations for the two children are likely to be similar, especially at school. The relative age effect is well evidenced within sport [12] and academic achievement [13]. It has also been studied with regard to child mental health problems [14]. There is no single accepted definition of younger relative age within the literature. Here we refer to children born at least in the younger half of the school year, however, others have defined this as the youngest one, two, three or 4 months of the year.

In relation to ADHD, the relative age effect has usually been demonstrated in countries with high prescribing rates for ADHD [15, 16], whereas findings from countries with lower prescribing rates have been mixed [2, 17, 18]. This area has important implications for diagnostic and prescribing practice as well as school entry policy. It has been argued that the relative age effect may represent the more immature behaviour of younger children being diagnosed and treated as ADHD and, therefore, more relaxed school entry policies may be able to offset this [15, 17]. This systematic review aims to investigate the strength of evidence for a relative age effect, distinguishing countries known to have higher and lower prescribing rates [19, 20]. It addresses three key questions—Is there an association between younger relative age, defined as being in the second half of the academic year, and: (1) the presence of high levels of ADHD symptoms, (2) receiving a clinical diagnosis of ADHD and (3) receiving medication for ADHD?

## Methods

A literature search was conducted with the assistance of an information specialist. This covered articles published from the 1st of January 2000 to the search date of the 7th September 2017. Databases searched included: MEDLINE, EMBASE, PsycINFO, Web of Science, ERIC, Psychology and Behavioural Sciences Collection and The Cochrane Library. Search keywords comprised: (1) Various terms for ADHD including: Attention Deficit Disorder with Hyperactivity, ADHD, ADDH, ADHS, hyperkinesis, hyperactive* and inattention* and (2) Relative age, relative maturity, relative immaturity, young for grade, young for year, old for grade or old for year. Additional studies were identified through checking reference lists of obtained articles. A further update search using the same search terms and databases was conducted on the 23rd of November 2017.

Abstracts were screened independently by JH and KS with 100% agreement, and then full text assessments were conducted by JH. All articles were available to download from online sources. Studies not published in English were translated by colleagues (*n* = 2) and assessed by JH.

### Inclusion criteria

Research articles were included which reported data from: a dimensional measure of ADHD symptoms, diagnoses or prescription provision amongst children or adolescents up to 18 years of age, where chronological age, including month (either reported as grouped months or actual month) of birth of participants, was recorded.

### Exclusion criteria

Papers were excluded for the following reasons: were case reports or conference abstracts; only data for individuals aged over 18 were used; no chronological age by month of birth data was recorded, and/or they focused on disorders or behaviour problems but did not specifically report on ADHD. Intervention studies were excluded unless they contained relative age comparison data.

### Analysis

Each study was assessed for bias using a modified version of the Newcastle–Ottawa assessment scale (NOS; [21]). The NOS scores a study based on its selection methods, comparability and outcome measures. Since the studies included in this review did not include an exposure, questions which related to this were excluded. A study could, therefore, score a minimum of 0 (low quality, high risk of bias) to a maximum of 6 (high quality, low risk of bias). Data were extracted and inputted into Review Manager version 5.3 for analysis. This review aimed to describe the literature and, where possible, conduct a quantitative analysis of the data via meta-analysis.

For the studies which met the inclusion criteria, data were extracted for the total number of children within each comparison group and the number of children who received a diagnosis of ADHD or ADHD medications. If a study met inclusion criteria but did not report the data in a format which could be included in the quantitative analysis, the authors were contacted to request the required figures.

Studies were divided based on country of origin, separating those from countries known to have higher rates of prescribing for ADHD (e.g. USA, Canada, Iceland and Israel), and those with lower rates (e.g. other European countries and Australia; [8]). For Germany, studies have reported prescribing prevalence rates ranging from 2.2% [22] to above 4% [20] and so, for the purposes of our analyses, will be treated as a high prescribing country. Where a study presented a number of comparisons, for example, children from the first month of the academic year and the last month as well as children from the first 4 months of the year compared to the last 4 months of the year, both comparisons were inputted into the analysis.

Due to high heterogeneity between studies, a random effects model using the Mantel–Haenszel method was used. Risk ratios (RR) with 95% confidence intervals were presented as the effect measure as this is the most commonly presented measure in studies.

## Results

A total of 123 references were retrieved through initial database searches and four through reference checking. A further two references were identified through the update search. After duplicates were removed, 63 abstracts were screened. Thirty records were excluded on the basis of: not relating to ADHD (9), response letters/reviews (4), case reports (5), no birth month information (8), adult data only (2) and tests of intervention (2).

The remaining 33 full-text articles were reviewed. A further 13 of these were excluded due to: no birth month information (1), ADHD not being separate from other child mental health disorders (1) and conference abstracts (11).

**Fig. 1:**
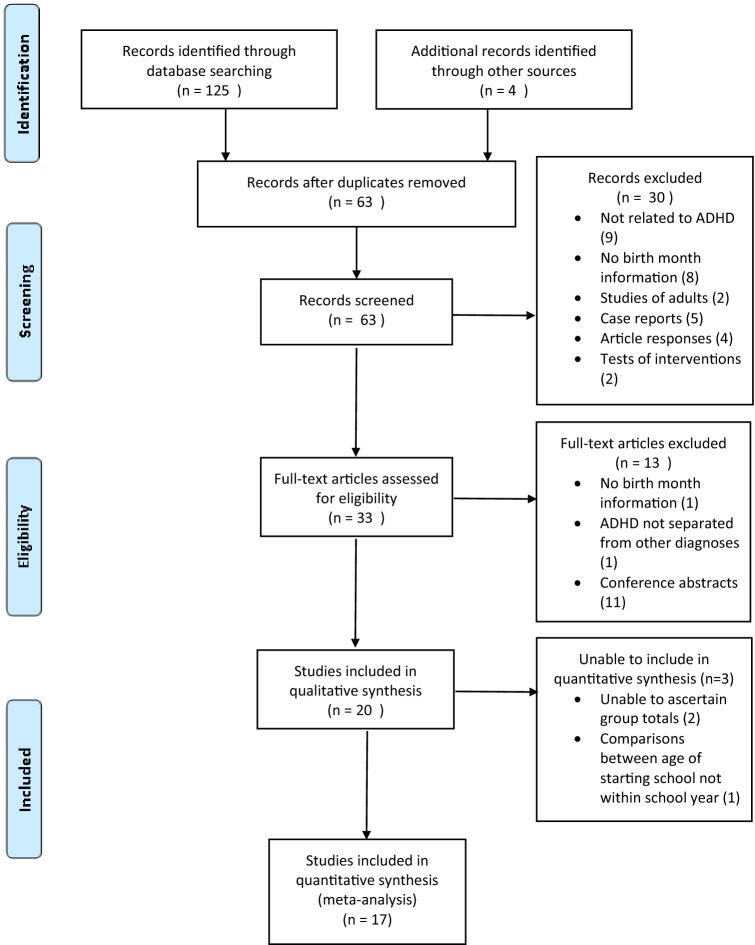
PRISMA flow diagram

Twenty studies were assessed for the review and data extraction, the characteristics of these studies are shown in Table 1. Six of these could not be included in the quantitative synthesis initially due to: information not being presented on the total number of children, with and without a diagnosis/medication receipt [8, 19, 22, 24, 25] or comparisons only being made between the starting school age not age within the school year [26]. However, following communications with the authors, data were provided for three studies [8, 20, 25] and were therefore included. The PRISMA flowchart for study selection is presented in Fig. 1.

**Table 1:** Characteristics of the studies included in this review

Study	Country	Data source	Sample size	Years studied	Ages studied	School starting age	Symptoms/diagnosis/medication	Symptom measures	Diagnosis definition	Medications definition	NOS and comments
Studies from countries with high prescribing rates											
Elder (2010) [27]	USA	Early Childhood Longitudinal study-Kindergarten Cohort	11784	1998–2007	Not stated	6 years of age. Date cut-off December 31st and August 31st, variable between states	Symptoms, diagnosis, medication	Parents’ reports, Teachers’ reports	Parents’ reports	Parent report-Methylphenidate, amphetamine based drugs	5
Evans (2010) [15]	USA	National health interview Survey (NHIS) Medical Expenditure Panel Survey (MEPS), Nationwide private healthcare company	NHIS 35343, MEPS 31641, Private 18559	1997–2006; 1996–2006; 2003–2006	7–17 years	6 years, cut-off date not specified	Diagnosis, medication	n/a	Self-report	Self-report and private insurance company claims Methylphenidate. Amphetamine	5
Hoshen (2016) [23]	Israel	Health insurance data, covers 50% of the population	1013149	2006–2011	6–17 years	The year in which the child turns 5, the date cut-off is variable in december based on lunar calendar	Medication	n/a	Prescriptions as a proxy for diagnosis	Prescriptions reimbursed amphetamine, methylphenidate, methamphetamine and atomoxetine	6 removed children born in November (youngest for year) from analysis due to high rates of holding back
Morrow (2012) [28]	Canada	Database information from Pharmanet, Medical Services Plan, Canadian Institute for Health information Discharge Abstracts Database	937943	2007–2008	6–12 years	Calendar year in which child turns 6 years of age	Medication	n/a	n/a	Methylphenidate, dextroampheamine, mixed amphetamine salts, atomoxetine	5
Schmiedeler (2015) [35]	Germany	Survey of 34 schools in in the area of Baden-Württemberg	928	Not stated	1st–4th school grades	Year during which child turns 6 years of age. Cut-off not stated	Not stated	Teacher reports	Reported by teachers	Reported by teachers	3
Schwandt (2016) [20]	Germany	Administrative medical claims records from all physicians registered with the social health insurance	7.2 million	2008–2011	4–14 years	Year during which child turns 6 years of age, date cut-off variable between states	Diagnosis, Medication	n/a	Database registered diagnosis	Methylphenidate or atomoxetine	6 Relative age effect present even with different date cut-offs. Extra data provided by authors
Zoega (2012) [16]	Iceland	Database of drug prescriptions	11785	2003–2009	Children born in 1994, 1995 and 1996	Calendar year in which child turns 6 years of age	Medication	n/a	Prescription used as a proxy for diagnosis	Amphetamine, methylphenidate, atomoxetine	6
Studies from other countries											
Chen (2016) [29]	Taiwan	Taiwan National Health Insurance Research Database	378881	1997–2011	4–17 years	September 1^st^-August 31^st^ academic year in which child turns 6 years of age	Diagnosis, Medication	n/a	ICD 9 code 314, given at least twice by board certified psychiatrists during follow up	Methylphenidate or atomoxetine	6 Relative age effect not seen in years 1998–1999, 2000–2001, 2002–2003, 2008–2009. Only seen in <12 year olds
Dalsgaard (2012) [17]	Denmark	Danish Psychiatry Central Register	416744	1990–2001	7 + years	Calendar year in which a child turns 7 years of age	Diagnosis	n/a	Registered diagnosis	n/a	6
Dalsgaard (2014) [8]	Denmark	Danish civil registration system	418396	1990–2001	7 + years	Calendar year in which a child turns 7 years of age	Medication	n/a	n/a	Dexamphetamine, methylphenidate or atomoxetine	6 Extra data provided by authors
Halldner (2014) [25]	Sweden	Swedish total population migrations and cause of death registers. National patient register. Prescribed drug register	56263 ADHD individuals, 10 random controls per case	2005–2009	6–69 years	Calendar year in which child turns 7 years of age	Symptoms, diagnosis, medication	Parent reported symptoms, selfreported symptoms	Coded in register or prescription as a proxy	Prescription in register Amphetamine, dexamphetamine, methylphenidate, atomoxetine	6 Extra data provided by authors
Karlstad (2017) [30]	Norway	Norwegian prescription database, Norwegian Patient Registry	509827	1998–2006	6–14 years	Calendar year in which child turns 6 years of age	Diagnosis, medication	n/a	Database diagnosis of ADHD either from specialist or GP	Methylphenidate, atomoxetine, racemic amphetamine, dexamphetamine, lisdexamphetamine	6
Krabbe (2014) [31]	The Netherlands	GP surgeries	2218	Not stated	5–12 years	October 1st-September 31st academic year in which child turns 5 years of age	Medication	n/a	Methylphenidate as a proxy for diagnosis	GP prescriptions. Methylphenidate only	4 Authors omitted all children born October and November from analysis to try and exclude those held back a year
Librero (2015) [32]	Spain	Health department Sistema de Informacion Poblacional	20237	Not stated	6–12 years	Calendar year in which child turns 6 years of age	Medication	n/a	Medication prescription used as a proxy for diagnosis	Methylphenidate, atomoxetine	5
Pottegard (2014) [18]	Denmark	The Danish National Prescription Registry, Danish Student Register, Danish Civil Registration System	932032	2000–2012	7–12 years	Calendar year in which child turns 7 years of age	Medication	n/a	n/a	Methylphenidate, atomoxetine, modafinil	6
Rivas-Juesas (2015) [33]	Spain	Retrospective case control study of patients referred to a neurology clinic	3469	1992–2012	Up to 15 years	Calendar year in which child turns 6 years of age	Diagnosis	n/a	Diagnosis based on assessment made in clinic	n/a	4
Folgar (2017) [34]	Spain	Sampled from Schools in Galacia	1547	Not stated	6^th^ grade of primary school and 1^st^ grade of secondary school	Calendar year when child turns 6 years of age	Diagnosis	n/a	Self-report	n/a	3
Studies not included in the meta-analyses											
Gokce (2016) [26]	Turkey	First and second grades of all public primary schools in Kadikoy county	3696	Not Stated	First and second school grades	60–66 months, cut-off date not specified	Diagnosis	n/a	Self-report	n/a	3 Not included. Comparison made dependent on age starting school, not relative age within school year
Sayal (2017) [19]	Finland	National Registers	6136	1998–2011	7–17 years	Calendar year in which child turns 7 years of age	Diagnosis	n/a	Individual registered within the Finnish Hospital Discharge Register with a diagnosis of ADHD	n/a	6 Not included. Data presented do not include the total number of participants per group
Whiteley (2017) [24]	Australia	Pharmaceutical Benefits Scheme	311384	2013	6–10, 11–15 years	The academic year from 1st July–30th June when the child turns 5 years	Medication	n/a	n/a	Not listed	5 Not included. No data published on group totals

For the three studies which included measures of ADHD symptoms, two showed evidence of a relative age effect. This was from symptom reports from teachers [27, 35] with weaker evidence of an effect from parents’ reports [35]. In contrast, the third did not show evidence of a relative age effect in parent-reported levels of symptoms [25]. A meta-analysis was not possible since the results were not directly comparable due to the use of different types of rating scales, e.g. Strengths and Difficulties Questionnaire, social rating scales and Autism–Tics, ADHD and other Comorbidities inventory.

For studies investigating the proportion of children receiving a diagnosis and/or medication, data were extracted and meta-analyses were conducted, separating studies into subgroups based on the outcomes studied (diagnosis or prescription) and whether the country was known to have higher or lower rates of ADHD prescribing. However, heterogeneity estimates were too high, for an overall analysis (diagnosis *I*^2^ = 97%, prescriptions *I*^2^ = 95%) to be presented.

**Fig. 2:**
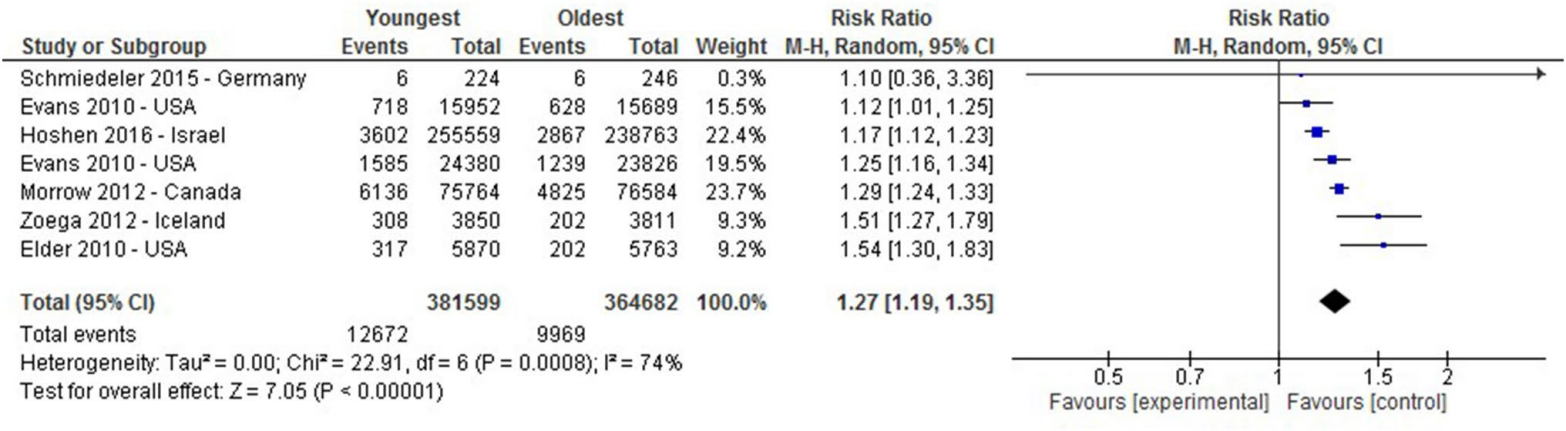
A Forest plot of studies comparing proportions receiving medication for ADHD between the oldest and youngest within the school year in higher prescribing countries. *Evans 2010 appears twice in this Figure due to presentation of data from Medical Expenditure Panel Survey (line 2) and Private Insurance claims (line 4) comparisons separately

**Fig. 3:**
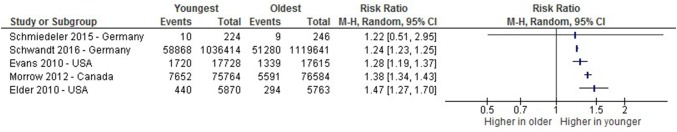
A risk-ratio plot of studies comparing proportions receiving a diagnosis of ADHD between the oldest and youngest within the school year in higher prescribing countries

A meta-analysis of the studies investigating the proportion of children receiving medication in higher prescribing countries showed a significant relative age effect with those younger in the academic year being more likely to receive medication for ADHD (*I*^2^ = 74%, RR 1.27 (1.19–1.35)), as shown in Fig. 2. However, the meta-analysis of studies reporting the proportion who received a diagnosis showed high heterogeneity (I^2^ = 91%) and therefore is not presented here, Fig. 3 shows the risk ratio from each study.

**Fig. 4:**
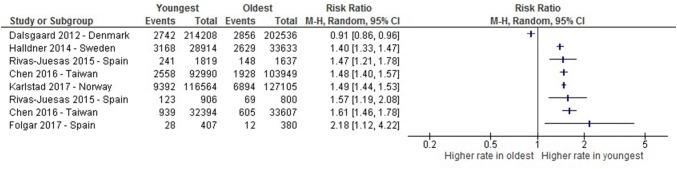
A risk-ratio plot of studies comparing proportions receiving a diagnosis of ADHD between the oldest and youngest within the school year in other countries. *Chen 2016 appears twice in this figure due to presenting comparison of the oldest ¼ of the year compared with the youngest ¼ (line 4) and the presentation of those born in the first month of the academic year and the last month (line 7). Rivas-Juesas 2015 appears twice in this figure due to data comparing the oldest 1/3 of the year compared with the youngest 1/3 (line 6) of the year and the oldest 6 months of the year compared to the youngest 6 months (line 3)

**Fig. 5:**
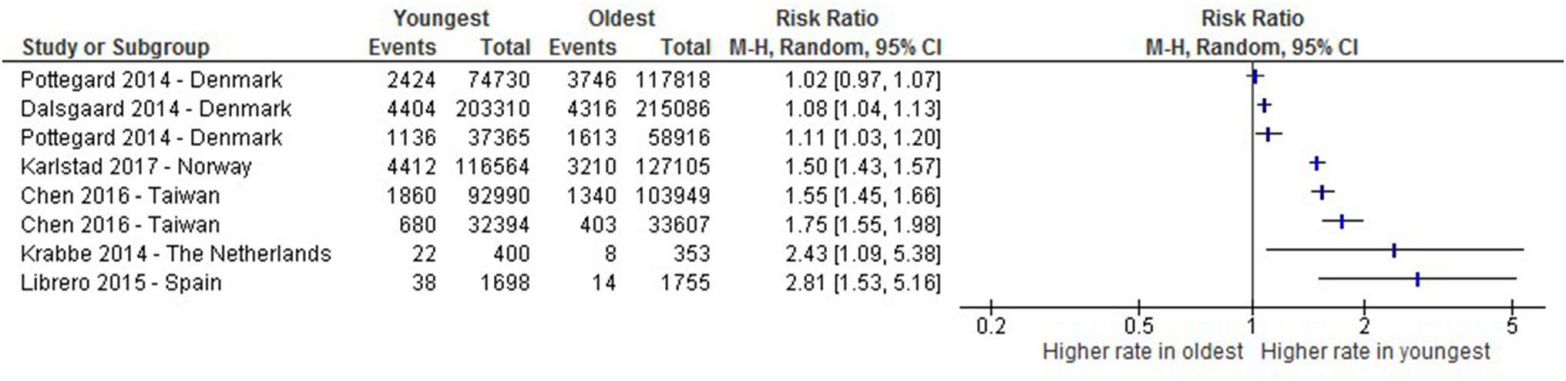
A risk-ratio plot of studies comparing proportions receiving medication for ADHD between the oldest and youngest within the school year in other countries. *Chen 2016 appears twice in this figure due to presented comparison of the oldest ¼ of the year compared with the youngest ¼ (line 5) and those born in the first month of the academic year and the last month (line 6). Pottegard 2014 appears twice in this figure due to presented comparisons of those born in the oldest 1 month and the youngest 1 month of the year (line 3) and the oldest 2 months compared with the youngest 2 months (line 1)

For the other countries, heterogeneity estimates were too high for reporting of the meta-analysis for either diagnosis or medication, *I*^2^ = 98% and *I*^2^ = 97%, respectively, as shown in Figs. 4 and 5.

As shown within the risk-ratio plots, almost all studies have shown, to some extent, higher proportions of ADHD diagnosis and prescriptions amongst the youngest in the academic year. The studies which did not show a significant relative age effect were from Denmark (RR 0.91, 95% CI (0.86–0.96); [17] and RR 1.02, 95% CI (0.97–1.07); [18]), and one from Germany (RR 1.55, 95% CI (0.51–2.95); [35])). However, there is variation in the magnitude of the risk ratio estimates between different studies, e.g. one finding that children born in the youngest month of the academic year were over 1.6 times more likely to be diagnosed with ADHD (RR 1.61, 95% CI (1.46–1.78)) and to receive medication (RR 1.75, 95% CI (1.55–1.98)), compared with the children born in the month after the cut-off [29]. In comparison, a study from Israel showed an increased risk ratio of around 1.2 for the youngest third of the school year, compared with the oldest third (RR 1.17, 95% CI (1.12–1.23) [23].

## Discussion

This review has found that the majority of studies show evidence of a relative age effect influencing both the diagnosis of and receipt of medication for ADHD. This was demonstrated most clearly within studies from higher prescribing countries, with a modest pooled risk estimate of 1.27 for medication amongst the youngest in the school year compared with their older peers in the same school year. Data from the other countries were more mixed, with high levels of heterogeneity.

Differences between study results might reflect methodological differences. As shown in Table 1, studies differed by sample size, years studied, ages studied and methods of reporting and recording ADHD diagnosis and medication. However, a number of other factors may contribute to the differences found across studies.

First, as noted above, there are significant variations in the estimated rates of ADHD diagnosis and medication provision between countries [8]. Schwandt and Wuppermann [20] plotted the relative effect sizes of the relative age effect against diagnosis prevalence rates. They found a positive correlation, with countries with higher overall prevalence rates showing a larger relative age effect, an effect which was also shown between different regions in Germany. This suggests the possibility of misdiagnosis in relation to children with young relative age. However, this explanation does not fit with findings from a study using data from Finland, a country with low diagnosis and prescription rates but with evidence of a relative age effect [19].

Second, linked to the above, there are differences in diagnostic practice across countries, for example, which professionals are able to give a diagnosis [15, 17]. The culture of diagnostic practice within a country’s health system may have an influence on the relative age effects found.

Third, school entry regulations and policy may play a role. Some studies have highlighted the possibility of children being held back an academic year as a possible factor influencing the magnitude of relative age estimations and contributing to heterogeneity [15, 17, 29]. Not only do countries differ in their age of school entry, but also in the extent to which these regulations are adhered to. For example, in Taiwan, children may possibly attend school 1 year early because of an arrangement between parents and teachers, purposefully rendering them the youngest [29]. In the US in the mid-1990s, around 10% of pupils delayed entry into kindergarten, this was more common for boys and for those with developmental delay [15]. In comparison, in Denmark, only 60% of children born in the last quarter before the cut-off date are enrolled in school for their assigned year [17]. In Israel, parents often opt or are recommended by the child’s kindergarten teacher to delay the commencement of school [23]. However, in the study from Iceland [16] just 0.7% of children were estimated to be either a year ahead or behind. If immaturity being mistaken for ADHD is a cause of higher rates of diagnosis and medication amongst those youngest in the year, encouraging greater awareness of this amongst parents, pre-school staff and clinicians may be useful in addressing the relative age effect. However, research is needed on the potential benefits and harms of holding children back a year, e.g. moving children to be the oldest within a year group could increase adults’ expectations of them [36, 37].

Fourth, teacher perceptions, in particular, may play a role. Elder [27] demonstrated that teachers’ ratings of ADHD symptoms showed a significant relative age effect, having a much greater magnitude than parents’ ratings. This suggests that teachers are more likely to compare children with others in the same school year rather than by chronological age and thus may contribute to the possible over-diagnosis of ADHD in younger children. In support of this finding, other studies have found no relative age effect in parental reports of symptoms or self-reported symptoms from adults with ADHD [25].

## Strengths and limitations

To our knowledge, this is the first systematic review of the association between relative age and ADHD. This review has identified and brought together existing research in a rigorous and systematic manner, enabling meta-analyses of the data, where possible. However, there are a number of potential limitations. First, since there are a number of different ways in which the relative age effect can be flagged within a study’s title and abstract there is a risk that some studies may have been missed. Second, some studies did not publish their data in a form which could be inputted to the meta-analysis. This meant that some large studies within the field could not be included in the meta-analysis [19, 24]. Others excluded children born in the first or last months of the academic year due to their likelihood of being held back [23, 31], this may have introduced bias within the data. Third, high levels of heterogeneity meant that it was not always possible to conduct a meta-analysis.

### Clinical and research implications

These findings have significant clinical implications. Since there is mounting evidence of a relative age effect on ADHD diagnosis and medication in most countries studied, which may imply possible misdiagnosis of relatively immature children, it is possible that some relatively young children may be unnecessarily offered and exposed to medication, the long-term effects of which are still not fully understood. When assessing for ADHD, clinicians should also bear in mind that teachers may be more likely than parents to apply same year-group peer referencing when completing rating scales [27].

In terms of educational implications, these findings should be considered in relation to school entry regulations. It may be that through more flexible school entry criteria, relatively immature children may be allowed more time to develop prior to entering schooling and potentially avoid unnecessary diagnosis and medication.

In terms of research, further work is needed to understand whether the relative age effect is due to misdiagnosis of younger children, for example through a longitudinal study showing whether these children continue to meet criteria for ADHD at later stages. Although some studies have explored the association between certain population characteristics and a relative age effect, further work is needed to explore the mechanisms under-pinning this effect. Family studies examining whether these relatively young children lack familiality of the disorder would also be useful. The literature to date has used epidemiological data. Qualitative research, in particular, could be useful in improving our understanding about the processes contributing to the relative age effect.

## Conclusions

This systematic review and meta-analysis has drawn together worldwide studies investigating the relative age effect in the symptoms, diagnosis and medication treatment of ADHD amongst children and adolescents. It has shown that the relative age effect is evident in the majority of countries, however, there is considerable variation in its magnitude. Possible explanations include: overall diagnostic rates, national differences in diagnostic practice and school entry regulations and the influence of different informant sources. In isolation, none of these theories are able to fully explain the differences shown. Further research is needed to better understand the reasons for the relative age effect. At an individual level it is crucial for clinicians to consider a child’s relative age when assessing for ADHD.
